# Recombinant growth hormone therapy in children with short stature in Abu Dhabi: a cross-sectional study of indications and treatment outcomes

**DOI:** 10.3389/fped.2025.1516967

**Published:** 2025-05-20

**Authors:** Sara Salem Al Jneibi, Fatima Taha, Marwa Hammouri, Zahraa Allami, Stefan Weber, Jamal Aljubeh, Sareea Al Remeithi

**Affiliations:** Department of Pediatrics, Division of Endocrinology, Sheikh Khalifa Medical City (SKMC), Abu Dhabi, United Arab Emirates

**Keywords:** growth hormone, short stature, recombinant, children, deficiency, endocrine

## Abstract

Early diagnosis of the pathological cause, if any, of short stature in children can lead to prompt intervention with recombinant growth hormone (rGH) treatment, potentially allowing them to achieve their true genetic height potential. However, it is crucial to identify children most likely to benefit from rGH treatment.

**Methods:**

This cross-sectional, retrospective study provides a broad overview of rGH prescribing patterns and evaluates both short- and long-term treatment outcomes in children treated at the Pediatric Endocrinology Clinic, Sheikh Khalifa Medical City, Abu Dhabi, UAE, between January 2011 and December 2022. One- and three-year outcome data for children treated with rGH for different diagnoses of short stature were assessed.

**Results:**

Idiopathic short stature (ISS) accounted for 34.8% of the cases for which rGH was prescribed. A significant response [mean height gain of ≥0.3 standard deviation score (SDS)/year] was seen across all assessed short-stature diagnoses, with the highest gain seen in the growth hormone deficiency (GHD) diagnosis group at the 1-year and 3-year treatment time points. More than 90% of the children diagnosed with GHD and ISS achieved normal final adult height. Younger age at rGH initiation, lower height SDS at baseline, and pre-pubertal status were associated with better outcomes post 1 and 3 years of rGH therapy. Greater response at 1 year of rGH therapy was associated with better final adult height outcome.

**Conclusions:**

ISS was the most common indication for which rGH was prescribed in this study. A favorable increment in the height SDS of the rGH-treated children during their 1- and 3-year follow-ups was observed. Age, pubertal status, baseline height SDS, and rGH response at 1 year were directly associated with significantly improved short- and long-term response to rGH treatment. These findings provide a broad overview of the baseline and therapeutic response characteristics of rGH-treated children with short stature in the UAE and can help in optimizing and personalizing treatment strategies.

## Introduction

Short stature, defined as “a condition in which the height of an individual is two standard deviations (SD) below the corresponding mean height of a given age, sex, and population group.” (1) Differentiating between the pathological and non-pathological etiologies of short stature is the key to determining the necessity and course of treatment. In most cases, familial genetic makeup and constitutional delay in growth and puberty are responsible for short stature (2). It may also be attributed to underlying endocrine disorders like growth hormone deficiency (GHD) and hypothyroidism. Several genetic disorders leading to short stature, such as Turner syndrome (TS), Noonan syndrome, and Prader-Willi syndrome (PWS), have also been identified (1, 2).

Given the limited window during which growth occurs, early identification of the underlying conditions can facilitate timely intervention, enabling children to achieve their full genetic height potential. Initially approved for treating GHD, recombinant growth hormone (rGH) is now approved for other conditions that negatively impact growth. These include TS, PWS, chronic renal disease (CKD), small for gestational age (SGA), Noonan's Syndrome, short stature homeobox-containing gene (SHOX) deficiency, and idiopathic short stature (ISS) (3, 4).

Although rGH has been approved for several indications, its use must be carefully targeted towards those most likely to benefit. The application of rGH for treating children with ISS remains somewhat controversial (5). The diagnosis of ISS, defined as short stature in the absence of any known endocrine, systemic, genetic, or nutritional defects, is primarily by exclusion. Children diagnosed with ISS represent a genotypically and phenotypically diverse group. Furthermore, a constitutional delay of growth and puberty is often a contributing factor in the diagnosis of ISS (6).

A study by Al-Abdulrazzaq et al. analyzed the pattern of use and outcomes associated with 1 year of rGH therapy in 60 children treated at a single hospital in Kuwait. The study identified GHD as the most common indication for prescribing rGH therapy. It also showed a significant 1-year response to rGH therapy in children diagnosed with GHD, SGA, and TS (and variants), but the response was not statistically significant in children diagnosed with ISS (7).

Our study aimed to get an overview of the rGH prescribing pattern and assess the short-term and long-term therapy outcomes among children treated with rGH at the Pediatric Endocrinology Clinic at our center, SKMC, Abu Dhabi, in UAE. Our primary objectives were to (1) ascertain the main indications for which rGH is prescribed, (2) determine the mean age at which rGH treatment is initiated and compare it with international recommendations, and (3) assess the short-term rGH treatment response, (measured as height gained after 1 and 3 years of therapy). The secondary objectives of this study were to (1) assess the long-term rGH treatment response upon reaching final adult height (FAH) and (2) investigate the potential predictors for favorable outcomes at 1 and 3 years of therapy.

## Methods

This cross-sectional retrospective study assessed the short—and long-term outcomes in children treated with rGH at the Pediatric Endocrine Clinic, Sheikh Khalifa Medical City (SKMC), Abu Dhabi, UAE, between January 2011 and December 2022.

### Inclusion and exclusion criteria

The inclusion criteria were children diagnosed with short stature and the availability of their 1-year and 3-year rGH treatment data. Children with chronic and active use of systemic steroids at baseline and those with missing baseline or missing 1-year treatment follow-up data were excluded from this analysis.

### GHD diagnosis

A diagnosis of GHD was established if a patient failed to pass at least one of two GH stimulation tests (combined sequential same-day tests: Clonidine/Arginine). Additional indicators included slow growth velocity for age, delayed bone age (BA), and low or low-normal insulin-like growth factor (IGF)-1 levels. Sex steroid priming was not performed before GH stimulation in this study. GHD was confirmed if the peak GH level was <7 ng/ml. The stringent cut-off of <7 ng/ml was chosen due to the use of advanced immunochemiluminescent and newer monoclonal-based assays at our center.

An MRI of the brain was conducted for all children diagnosed with GHD before initiating GH therapy. While most children with constitutional delays in growth and puberty exhibited normal growth velocity and strong family history indicators, GH stimulation was performed in those with subnormal growth velocity and/or severe short stature to rule out GHD.

Relevant data such as patient demographics, anthropometric measurements [height standard deviation scores (SDS), BMI SDS, growth velocity], puberty status, bone age (BA) at baseline, indications for rGH administration and doses administered, mid-parental height (MPH) and FAH were extracted from the electronic medical records and analyzed statistically.

The study was approved by the ethical committee of Sheikh Khalifa Medical City, Abu Dhabi, UAE. Statistical analysis of the data was conducted using Stata version 17, StataCorp LLC, Texas, USA.

The short-term response of the rGH therapy was evaluated using Bang and Ranke criteria by calculating the difference between the height SDS at 1 year or 3 years and the height SDS at baseline. A height gain of ≥ 0.3 SDS per year was considered a “good” response (5, 8).

The long-term response of rGH therapy was assessed from the FAH SDS of the study participants. An FAH SDS of ≥−2 (corresponding to a height of ≥ 162.5 cm in males and ≥ 150.3 cm in females according to CDC estimates) was categorized as a “good/acceptable” response, and an FAH SDS of <−2 was graded as a suboptimal ‘ response. Participants achieving an FAH of ≥ −2 SDS were categorized as having ‘normal’ FAH.

For statistical inferences, the Stata 18 packages were used (StataCorp, College Station, Texas, USA). Mean, median, and standard deviations were calculated using the package modules. Discrete variables were tested using Pearson's Chi-Square, and continuous variables were compared using a *t*-test. Uni- and multivariable analytics were performed using the same package, setting the *p*-value at 0.05 significance.

## Results

### Baseline characteristics and mean age at rGH treatment initiation

**Figure 1:**
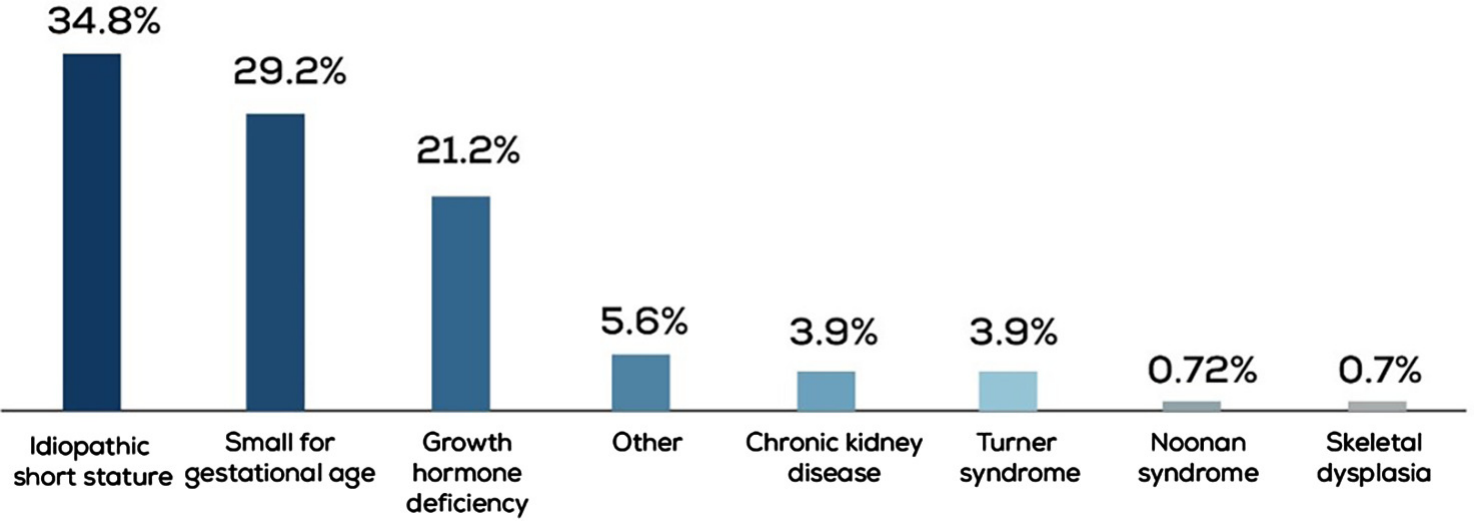
Indications for which rGH therapy was prescribed to the study group.

Data from 414 children treated for short stature between 2011 and 2022 were obtained. ISS was the most common diagnosis in 34.8% of the cases, followed by SGA and GHD in 29.2% and 21.2% of the cases, respectively (Figure 1). In the GHD cohort, 27.7% (*n* = 23) of patients had a peak GH level of <5 ng/ml.

Of the 414 children screened, 394 were identified as rGH-treatment naïve at baseline. Based on the inclusion criteria, 370 rGH-treated children were selected for further analysis. The children were stratified based on their diagnosis (GHD, ISS, SGA, TS, or CKD). Their auxological characteristics at baseline are shown in Table 1. The majority of the children diagnosed with Turner syndrome (TS) had a 45, X karyotype (*n* = 8), while four had an isochromosome, and the remaining four exhibited mosaicism. Children diagnosed with Prader-Willi syndrome, Kabuki syndrome, and Silver-Russel syndrome were categorized as “Other.”

**Table 1:** Auxological characteristic of the study population at baseline based on diagnosis (*n* = 370).

Variable	GHD *n* = 83	ISS *n* = 142	SGA *n* = 116	TS *n* = 16	CKD *n* = 13	*P*-value
Age at GH initiation in years, Mean ± SDS	10.02 (±3.6)	11.1 (±2.7)	8.9 (±3.2)	9.2 (±3.2)	6.5 (±3.2)	<0.0001
Sex, Male *n* (%)	57 (68.7%)	99 (69.7%)	68 (58.6%)	NA	9 (69.2%)	NA
Pre-pubertal, *n* (%)	64 (77.1%)	99 (69.7%)	90 (77.5%)	13 (81.2%)	8 (61.5%)	0.02
Height SDS, Mean ± SDS	−2.7 (±0.6)	−2.5 (±0.47)	−2.7 (±0.63)	−3.3 (±0.8)	−3.3 (±0.98)	<0.0001
BMI SDS, Mean ± SDS	−0.48 (±1.5)	−0.99 (±1.2)	−1.6 (±1.6)	0.1 (±1.1)	−0.43 (±0.9)	<0.0001
Bone age in years, Mean ± SDS	7.62 (±3.32)	8.83 (±3.30)	7.2 (±3.55)	8.25 (±2.57)	4.58 (±2.86)	<0.0001
MPH SDS, Mean ± SDS	−1.2 (±0.70)	−1.3 (±0.61)	−1.4 (±0.80)	−0.79 (±0.78)	−1.1 (±1.03)	0.02
Growth velocity, cm/year Mean ± SDS	4.7 (±1.6)	5.4 (±1.8)	5.2 (±1.5)	4.7 (±1.1)	3.7 (±1.2)	0.0047
GH dose, (mg/kg/day) Mean ± SDS	0.04 (±0.01)	0.048 (±0.01)	0.047 (±0.01)	0.05 (±0.002)	0.048 (±0.01)	<0.0001

GHD, GH deficiency; ISS, Idiopathic short stature; SGA, small for gestational age; TS, Turner syndrome; CKD, Chronic kidney disease.

**Figure 2:**
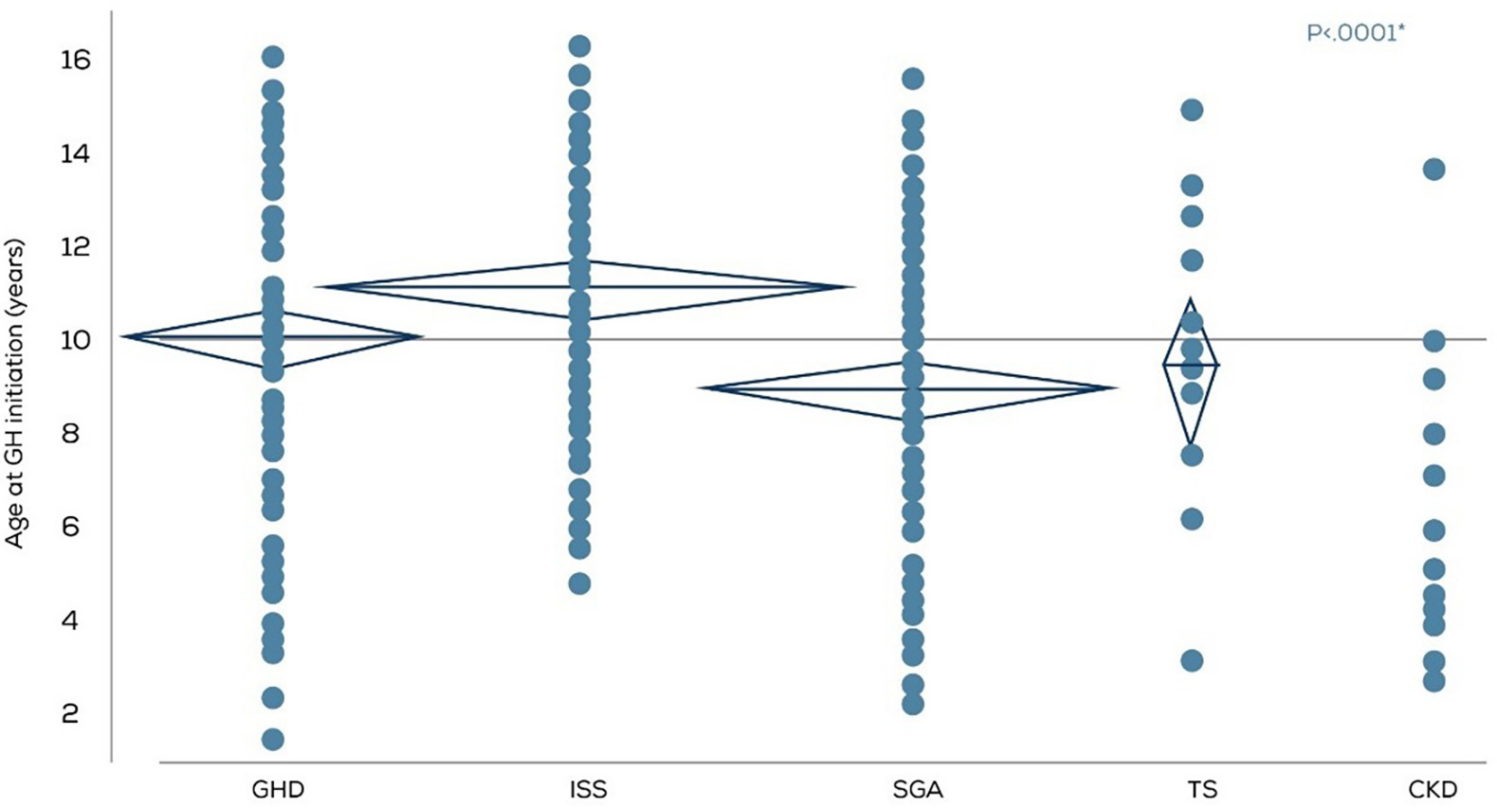
Age in years at rGH therapy initiation according to the diagnosis (*n* = 370). GHD, GH deficiency; ISS, Idiopathic short stature; SGA, small for gestational age; TS, Turner syndrome; CKD, Chronic kidney disease.

The mean age at which rGH therapy was initiated varied according to the diagnosis (Figure 2). The rGH therapy was started earlier, at a mean age of 6.5 years in the CKD group, and much later in the ISS group at a mean age of 11.1 years. However, it should be noted that compared to 142 children in the ISS diagnosis group, the CKD cohort had only 13 children. For GHD, SGA, and TS, the mean age for rGH therapy initiation ranged between 8 and 10 years.

### Short-term rGH response

**Figure 3:**
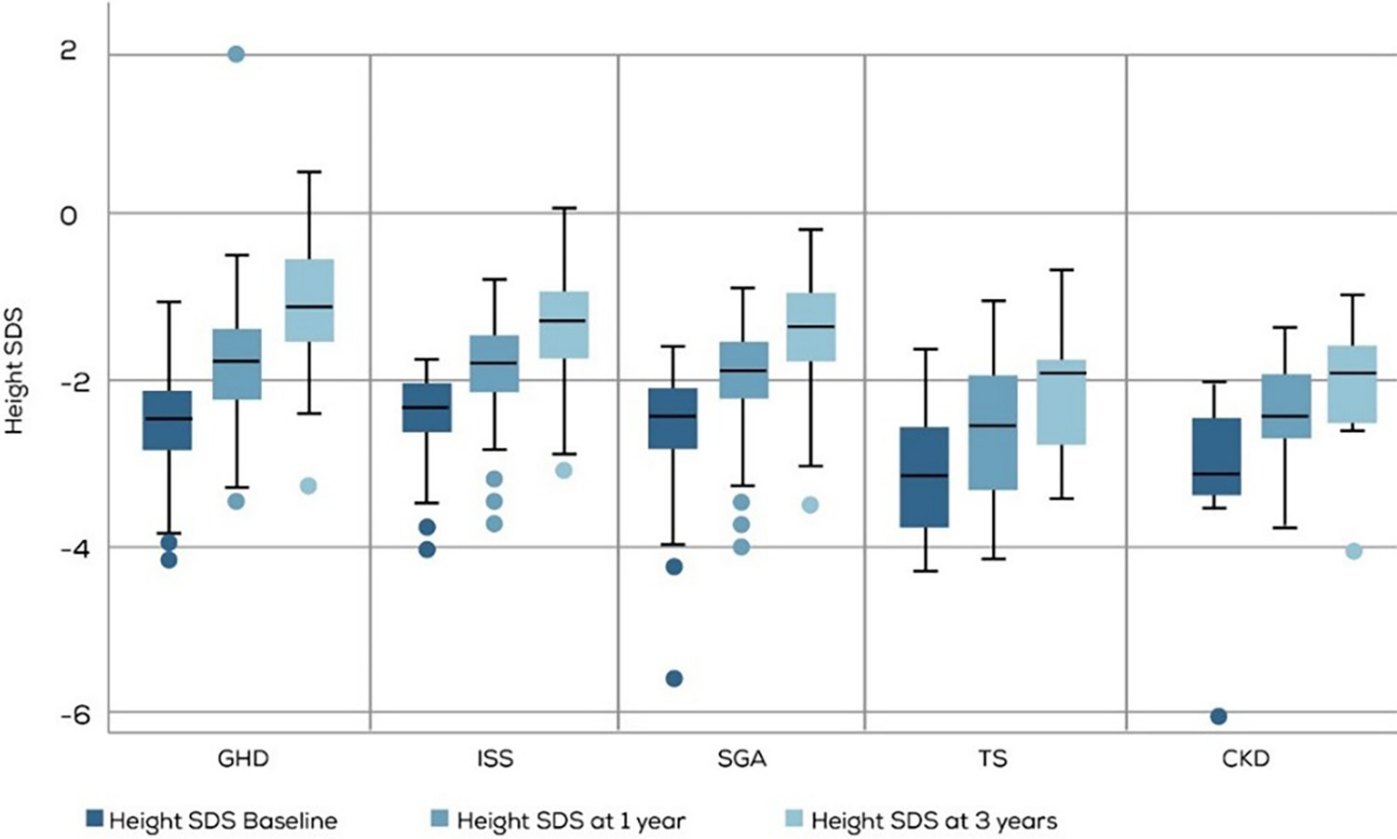
Height SDS at baseline, year-1 and year-3 of rGH therapy. GHD, GH deficiency; ISS, Idiopathic Short stature; SGA, small for gestational age; TS, Turner syndrome CKD, Chronic kidney disease.

Short-term rGH response was evaluated by calculating the height gained after 1 year and 3 years of therapy (Table 2 and Figure 3). A significant response, defined as a mean height gain of ≥0.3 SDS/year, was seen across all diagnoses, with the highest gain seen in the GHD group at both the 1-year and 3-year treatment time points. Similarly, the mean growth velocity (cm/year) was the highest in the GHD group compared to other groups at both the tested time points. In this context, it should be noted that the number of children diagnosed with TS and CKD used for this analysis was relatively lower than those diagnosed with GHD, ISS, and SGA.

**Table 2:** Short-term treatment outcomes after 1 year and 3 years of rGH treatment.

Treatment response at 1 year					
Variable	GHD *n* = 83	ISS *n* = 142	SGA *n* = 116	TS *n* = 16	CKD *n* = 13
Height SDS at baseline, Mean ± SDS	−2.7 (±0.6)	−2.5 (±0.47)	−2.7 (±0.63)	−3.3 (±0.8)	−3.3 (±0.98)
Ht SDS at 1 year, Mean ± SD	−1.9 (±0.75)	−1.9 (±0.51)	−2.1 (±0.60)	−2.7 (±0.97)	−2.6 (±0.69)
Ht SDS Gain, Mean ± SD	0.72 (±0.74)	0.58 (±0.31)	0.59(±0.37)	0.56 (±0.30)	0.67 (±0.58)
GV (cm/year) Mean ± SD	9.6 (±2.2)	9.1 (±1.8)	8.8 (±1.6)	7.9 (±1.9)	8.4 (±2.2)
Significant responders^a^ *n* (%)	71 (85.5%)	121 (85.2%)	95 (81.9%)	13 (81.2%)	10 (76.9%)
Treatment response at 3 year					
Variable Total *n*=	GHD *n* = 64	ISS *n* = 82	SGA *n* = 68	TS *n* = 15	CKD *n* = 9
Height SDS at baseline, Mean ± SDS	−2.5 (±0.47)	−2.7 (±0.63)	−3.3 (±0.8)	−3.3 (±0.98)	−2.7 (±0.6)
Ht SDS at 3 years, Mean ± SD	−1.2 (±0.8)	−1.4 (±0.6)	−1.5 (±0.7)	−2.2 (±0.8)	−2.3 (±0.9)
Ht SDS gain at 3 years, Mean ± SD	1.6 (±0.7)	1.1 (±0.4)	1.2 (±0.6)	1.1 (±0.4)	1.1 (±0.6)
GV (cm/year) Mean ± SD	7.5 (±2.4)	6.5 (±1.9)	6.6 (±1.6)	6.3 (±1.8)	5.9 (±2.5)
Significant responders^a^ *n* (%)	53 (82.8%)	62 (74.6%)	54 (79.4%)	10 (66.6%)	7 (77.7%)

GHD, GH deficiency; ISS, Idiopathic short stature; SGA, small for gestational age; TS, Turner syndrome; CKD, Chronic kidney disease.

^a^ Defined as a gain of ≥0.3 SDS of height per year (8).

### Long-term rGH treatment response

**Figure 4:**
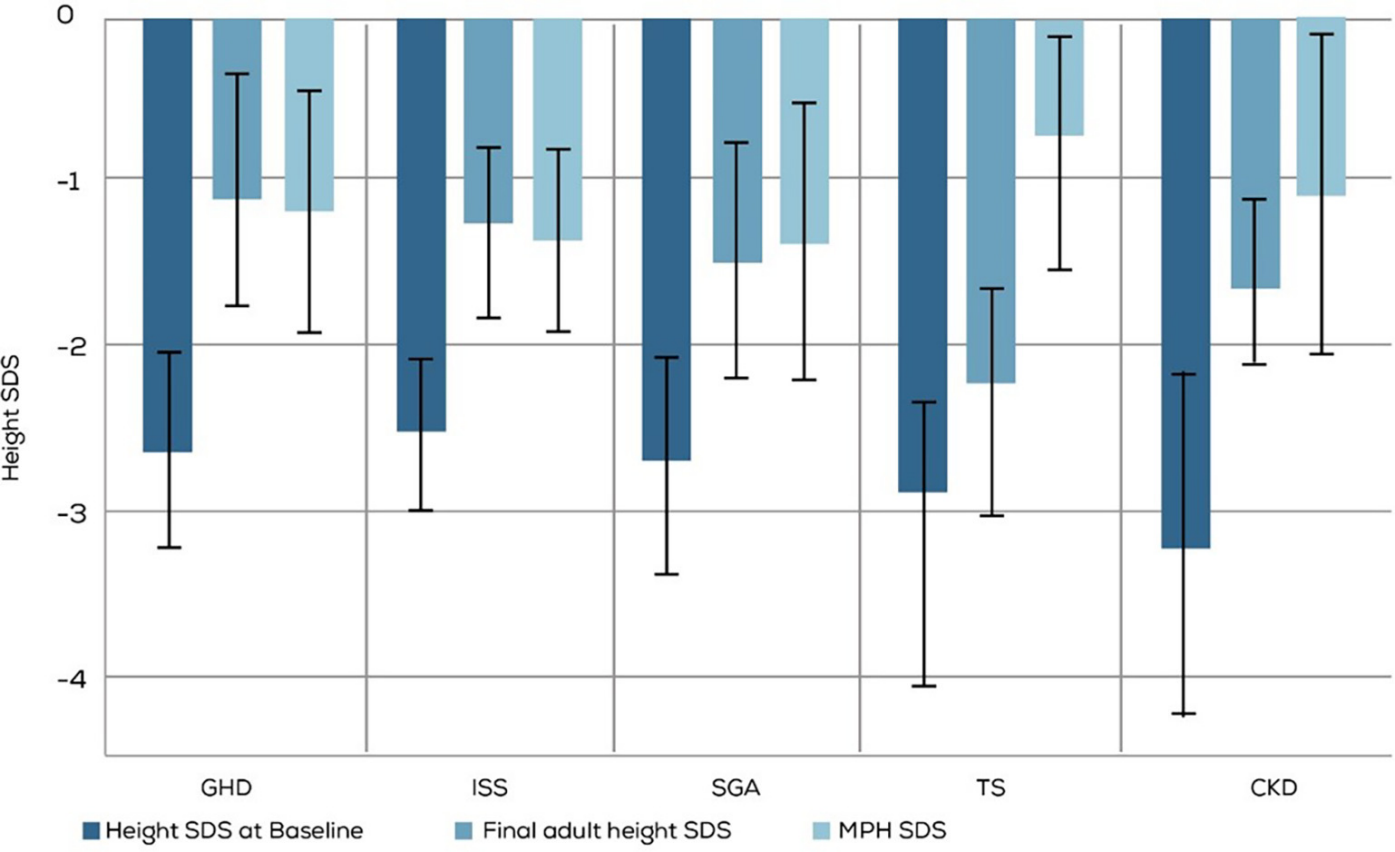
Comparison of baseline height SDS and MPH SDS with long-term rGH treatment outcomes assessed by FAH SDS. GHD, GH deficiency; ISS, Idiopathic Short stature; SGA, small for gestational age; TS, Turner syndrome; CKD, Chronic kidney disease. MPH, mid parenteral height.

The final adult height outcomes of 153 out of 370 treated children were available and used for this analysis (Table 3 and Figure 4). The mean FAH SDS ≥ −2, which was considered a good/acceptable response, was seen in children with all diagnoses except TS. Moreover, more than 90% of the children diagnosed with GHD and ISS achieved normal FAH (SDS ≥ −2).

**Table 3:** Long-term rGH treatment outcomes assessed by FAH SDS (*n* = 153).

Variable	GHD *N* = 35	ISS *N* = 61	SGA *N* = 42	TS *N* = 11	CKD *N* = 4
Height SDS at baseline Mean ± SDS	−2.7 (±0.6)	−2.5 (±0.47)	−2.7 (±0.63)	−3.3 (±0.8)	−3.3 (±0.98)
FAH SDS Mean ± SDS	−1.15 (±0.65)	−1.3 (±0.60)	−1.5 (±0.70)	−2.3 (±0.73)	−1.6 (±0.51)
Duration of therapy in years; Mean ± SDS	4.95 (±1.61)	4.25 (±1.81)	4.93 (±1.99)	4.90 (±1.93)	8.41 (±1.93)
MPH SDS Mean ± SDS	−1.2 (±0.70)	−1.3 (±0.61)	−1.4 (±0.80)	−0.79 (±0.78)	−1.1 (±1.03)
% achieved normal FAH (SDS ≥ -2SD)	91.4%	90.2%	73.8%	45.5%	75.0%

GHD, GH deficiency; ISS, Idiopathic Short stature; SGA, small for gestational age; TS, Turner syndrome; CKD, Chronic kidney disease. FAH, final adult height.

### Clinical predictors of improved short-term and long-term rGH treatment response

Multiple parameters — diagnosis/indication, age at rGH therapy initiation, rGH response post-1-year treatment, gender, puberty status at baseline, height SDS at baseline, BMI SDS at baseline, bone age (BA) status, and MPH SDS — were evaluated to identify potential predictors of improved response to rGH therapy. Among the tested parameters, younger age at rGH initiation, lower height SDS at baseline, and pre-pubertal status were associated with better outcome to rGH therapy (Table 4). Apart from these factors, a greater response after 1 year of rGH therapy and duration of therapy were associated with a better FAH outcome (Table 5).

**Table 4:** Multi-regression analysis: clinical predictors for a significant response post 1 and 3 years of therapy.

Variable	At 1 year *P* value (Coefficient)	At 3 year *P* value (Coefficient)
Age of initiation	0.017 (−0.06)	0.591 (0.02))
Height SDS at baseline	<0.0005 (0.98)	<0.0005 (0.80)
Puberty status at baseline	0.016 (0.16)	0.159 (0.15)
BMI SDS at baseline	0.134 (0.03)	0.45 (−0.03)
Duration of therapy (years)	0.476 (0.018)	0.073 (0.086)

**Table 5:** Multi-regression analysis: clinical predictors for better FAH outcomes.

Variable	At FAH *P* value (Coefficient)
Age of initiation	0.002 (−0.47)
Height SDS at baseline	<0.0005 (−2.08)
Puberty status (Pre-pubertal)	0.006 (1.05)
GH response at 1 year	<0.001 (2.05)
Duration of therapy (years)	0.03 (−0.358)

## Discussion

Significant heterogeneity exists in regulatory approvals of growth hormone replacement therapy in different countries, influencing physician prescribing patterns. Also, the variation in the quality of primary care and availability of genetic testing affect diagnoses for short stature and subsequent referrals to secondary care. These differences were highlighted by the two large studies from Europe and the US — NordiNet IOS (a multicenter, longitudinal, observational cohort study on data collected on Norditropin® using a web-based system) and ANSWER (a post-marketing registry of Norditropin® that was later converted to a noninterventional observation study). Both studies identified GHD as the most common indication for prescribing Norditropin®. SGA, TS, and ISS were the other indications for which Norditropin® was commonly prescribed, with SGA being more common than ISS in the European NordiNet IOS and ISS being more common than SGA in the US-based ANSWER study (9).

**Figure 5:**
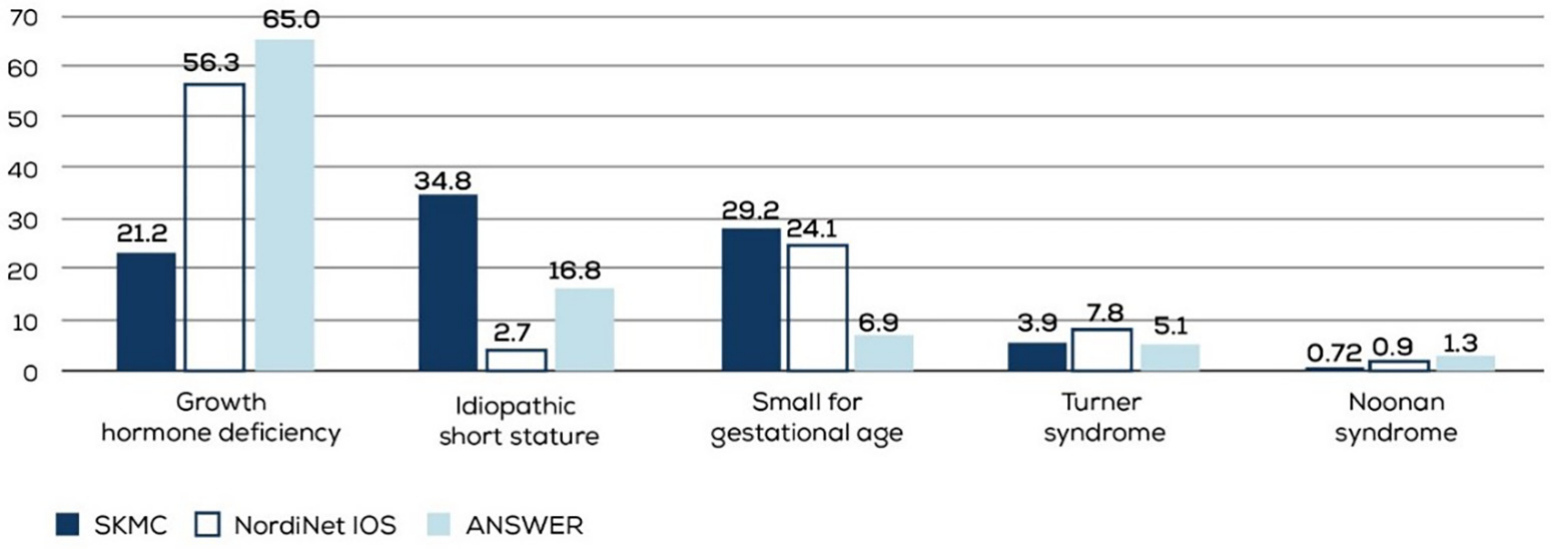
Indications for which rGH was commonly prescribed in the NordiNet IOS, ANSWER, and our study populations. SKMC, Sheikh Khalifa Medical City; NordiNet IOS, NordiNet International Outcome Study; ANSWER, American Norditropin Studies, Web-Enabled Research Program.

In our pediatric endocrinology clinic, ISS was the most common indication for rGH therapy, followed by SGA and GHD (Figure 5). ISS represents a heterogeneous group of patients, potentially involving underlying genetic etiologies that remain yet to be identified. Cultural factors, such as a high incidence of consanguinity and the distinct genetic profile of the region, may contribute to the increased prevalence of ISS diagnoses compared to GHD. Furthermore, the availability of comprehensive healthcare services and broad insurance coverage may facilitate earlier identification and management of ISS. This reflects a systemic pattern rather than a limitation, supporting the validity and relevance of our findings within our healthcare setting.

Confirming several observations from previous studies and those from the NordiNet IOS and ANSWER (9), we found more boys in our study population across all diagnoses. This gender imbalance is probably due to sociocultural pressure leading to more short-statured boys than girls being referred to specialists.

Most patients, over 75% of those diagnosed with GHD, SGA, and TS and over 65% of those diagnosed with ISS, were pre-pubertal when rGH therapy was initiated. The mean age at rGH initiation was the highest in the ISS diagnosis group. Height velocity in healthy children is age-dependent, and studies have shown children are more likely to achieve higher height velocity in the first year of rGH therapy if it is started at a young age (10). A study by Ranke et al. to identify factors likely to affect rGH outcome in children diagnosed with ISS found that younger age was associated with better first-year response to treatment (11). Age at which rGH therapy is initiated has also been shown to favorably affect growth response in children diagnosed with GHD, SGA, and TS (12).

**Figure 6:**
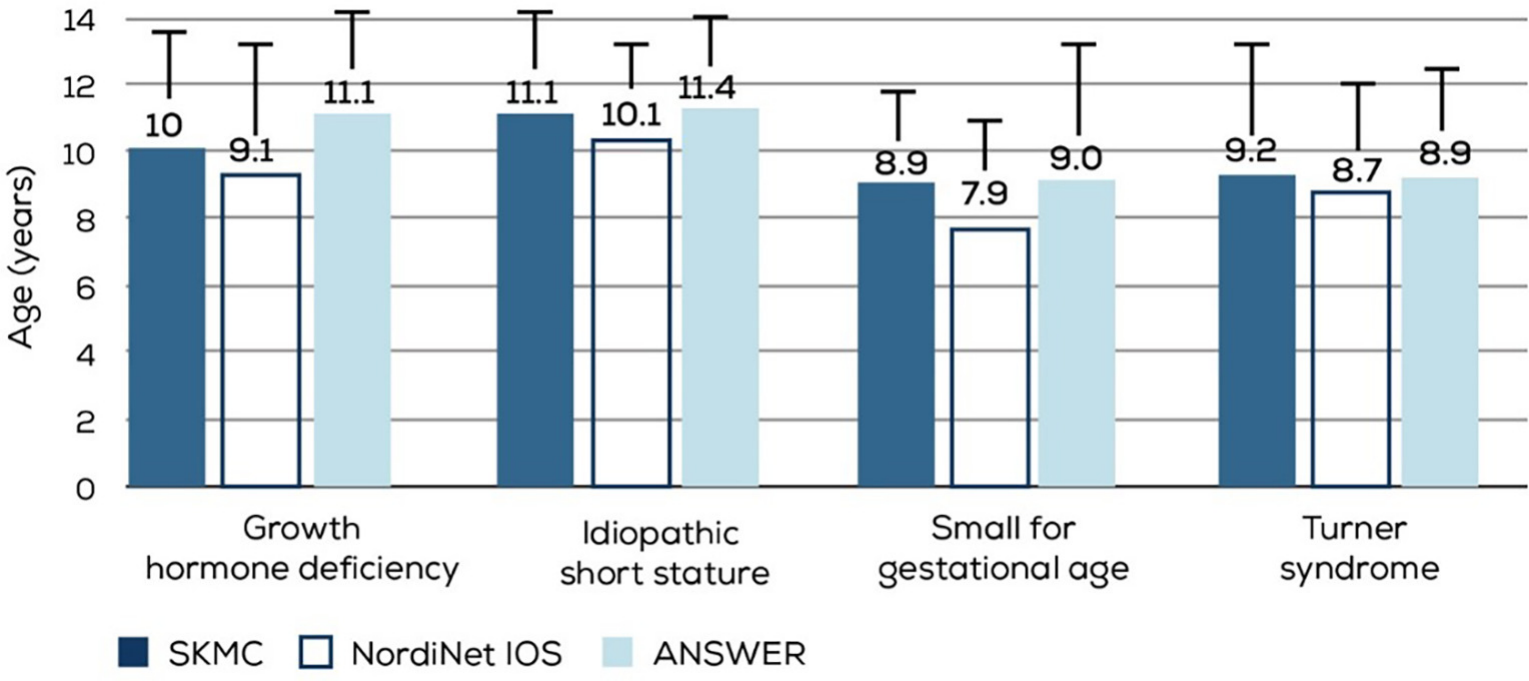
Comparison of mean age at rGH initiation in different diagnosis groups from the NordiNet IOS, ANSWER, and our study populations. SKMC, sheikh khalifa medical city; nordiNet IOS, NordiNet international outcome study; ANSWER, American Norditropin studies, Web-enabled research program.

The mean age of rGH therapy initiation across the four major diagnosis groups (GHD, SGA, TS, and ISS) was similar between our study population and the NordiNet IOS and ANSWER study populations (Figure 6) (9). However, the age at which rGH therapy was initiated is much higher than that recommended for SGA (>2 years in the US, > 3 years in Japan, and >4 years in Europe) and TS (4–6 years) (13, 14). A survey of 450 pediatricians and family medicine physicians from the Arabian Gulf region (KSA, UAE, Kuwait, Oman, Qatar, and Bahrain) has identified several gaps in short stature assessment, leading to delayed referrals (15). Reasons contributing to such sub-optimal diagnostic and referral processes need to be investigated. Additionally, steps must be taken to ensure children are appropriately and timely diagnosed, referred, and treated.

At 1-year and 3-year follow-ups, the mean height increment in all the diagnosis groups was ≥0.3 SDS, which qualified as a good response. Moreover, more than 80% of the children with all diagnoses except CKD showed a significant response to rGH treatment after 1 year, and almost 75% of the children with all diagnoses except TS showed a significant response to rGH treatment after 3 years of treatment. The highest response to rGH treatment after 1 and 3 years was seen in the children diagnosed with GHD, hinting that rGH may be more effective in GHD conditions than non-GHD conditions. Studies have also shown that children with severe GHD respond better to rGH than those with milder deficiency (5). Whether a similar trend exists in our study population needs further evaluation.

The target mean FAH of more than −2 SDS was observed in all the diagnosis groups except TS. Less than half of the children with TS reached the target FAH SDS. Also, a greater percentage of children diagnosed with GHD and ISS than those diagnosed with SGA and CKD achieved the target FAH SDS. Multiple factors like the dose, age at which therapy is initiated, and its duration affect growth response to rGH in TS. Favorable growth outcomes are observed when rGH therapy is initiated before the age of 4 years (5). Apart from other factors, the sub-optimal response to rGH in TS observed in our study could be because the therapy was initiated late; the mean age of therapy initiation in our study was 9.2 years.

Growth disorders manifest in a continuum, ranging from severe to mild GH deficiency at one end to mild to severe GH insensitivity at the other, with short stature due to idiopathic etiologies lying somewhere in between (16). Thus, it is not surprising to observe substantial variability in response to rGH therapy. The pleiotropic effects of the growth hormone on various physiological processes also add to rGH response variability. Although challenging, it is vital to identify parameters that can predict favorable outcomes and assist in making critical therapy-related decisions, like how long to continue treatment and dosage to use.

Previous studies have identified several predictors for favorable rGH response in children diagnosed with ISS, such as first-year growth response, younger age at therapy initiation, the difference between baseline and target height, and GH dose (17). An analysis of the data from the ANSWER program registry identified height velocity at 4 months, baseline age, baseline height SDS, baseline BMI SDS, and baseline IGF-I SDS as significant predictive factors for rGH response in children diagnosed with GHD (17).

Clinical predictors for a significant short-term (after 1 and 3 years of therapy) rGH response across all diagnoses in our study were — the age at rGH therapy initiation, height SDS at baseline, and puberty status at baseline. The younger the age at rGH therapy initiation and pre-pubertal status were associated with a significantly greater response to therapy. In addition to the above factors, the rGH response at 1 year and duration of therapy were also found to predict long-term response to therapy. Other clinical parameters that were evaluated — the indication for which rGH was prescribed, gender, BA status, and MPH SDS — did not influence short-term or long-term treatment response in our cohort.

## Conclusions

Compared to GHD reported in most other studies, ISS was the most common indication for which rGH was prescribed at our center. Irrespective of the diagnosis, we noticed a favorable increment in the height SDS of the rGH-treated children during their 1- and 3-year follow-ups. Age, pubertal status, baseline height SDS, and rGH response at 1 year were directly associated with significantly improved short- and long-term response to treatment.

The strengths of this study include a relatively large sample size and long-term follow-up through to adult height. While this study provides valuable insights into rGH prescribing patterns and treatment outcomes, limitations and some potential biases and confounders must be considered. As this is a single-center retrospective study, the results might not necessarily be applicable to be generalized to a larger scale, which represents a key limitation.

Referral and diagnosis biases may have played a role, as most children were referred by a primary care physician who was aware of the center's treatment options or by a family/friend member who experienced a similar condition. In such cases, many other short stature cases might be overlooked if they happen to be in a limited-resource area. Additionally, age at rGH therapy initiation and pubertal status significantly impact growth outcomes, making it difficult to determine the true effect of rGH across different diagnosis groups.

Lastly, long-term outcomes were assessed in a limited subset of patients, necessitating further research with larger, multicenter cohorts to validate these findings and improve our understanding of rGH treatment effectiveness in children with short stature. Although additional studies are required to further validate and extend our findings, they provide a broad overview of the baseline and therapeutic response characteristics of rGH-treated children with short stature in the UAE. Closing the gaps in the diagnosis and referral processes and identifying children most likely to respond to rGH therapy using clinical predictors can help optimize outcomes in rGH-eligible children from the UAE. Moreover, existing tools, such as digitally generated adherence reports, could be utilized to evaluate rGH therapy outcomes, offering real-world insights to help personalize dosing and treatment strategies.

## Data Availability

The original contributions presented in the study are included in the article/Supplementary Material, further inquiries can be directed to the corresponding author.
